# Opinion: The optimal use of risk factors to guide palivizumab prophylaxis against severe respiratory syncytial virus infection in moderate-to-late preterm infants

**DOI:** 10.3389/fped.2024.1343960

**Published:** 2024-01-12

**Authors:** Bosco Paes, Marcello Lanari, Barry Rodgers-Gray, John Fullarton, Xavier Carbonell-Estrany

**Affiliations:** ^1^ Department of Pediatrics (Neonatal Division), McMaster University, Hamilton, ON, Canada; ^2^ Paediatric Emergency Unit, IRCCS-Policlinico Ospedaliero-Universitario di Bologna, Bologna, Italy; ^3^ Violicom Medical Limited, Aldermaston, United Kingdom; ^4^ Neonatology Service, Hospital Clinic, Barcelona, Spain

**Keywords:** respiratory syncytial virus, risk factors, pediatric hospitalization, passive immunoprophylaxis, preterm infants

## Introduction

Respiratory syncytial virus (RSV) is the predominant viral pathogen associated with lower respiratory tract infection (LRTI) in young children (<5 years), causing 3.6 million hospitalizations (RSVHs) and 101,400 deaths annually worldwide (1). Moderate-to-late preterm infants (32–35 weeks' gestational age; wGA) are recognized to be at greater risk of severe RSV-LRTI (2, 3) and, for many countries, passive immunoprophylaxis with palivizumab remains the only preventive therapy available (4). To target palivizumab prophylaxis cost-effectively at moderate-to-late preterm infants who are at highest risk for serious RSV infection, several Risk Scoring Tools (RSTs) and predictive models have been developed incorporating social, demographic and environmental factors that determine risk for RSVH (5–12). Whilst there are several risk factors common to these RSTs and models, the number and definition of these variables and how they are scored to classify an infant's RSVH risk can vary considerably (Table 1). This leads to the question of what are the key risk factors that predict RSVH in moderate-to-late preterm infants and whether there is a preferred country-specific RST to endorse. Herein, we summarize the key attributes of an ideal RST and make the case for widespread adoption of the International RST (IRST) (5).

**Table 1:** Comparison of risk factor-guided approaches to identify moderate-to-late preterm infants at increased risk of RSVH.

RST (reference)	IRST^†^ (5)	FLIP-2 (7)	RISK (9)	RISK-II (10)	PONI (11)	FLIP (6)	CRST (8) [PICNIC]	SIN_LAZIO_ score (12)
Country	International^a^	Spain	Netherlands	Netherlands	International^d^	Spain	Canada	Italy
Source data	Pooled dataset of 6 prospective observational cohort studies (*n* = 13,475)	Prospective observational cohort study (*n* = 5,441) (13)	Prospective observational 2-cohort study (*n* = 2,421)	Prospective observational cohort study (*n* = 1,564)	Prospective observational cohort study (*n* = 2,390)	Prospective case-control study (*n* = 554) (14)	Prospective observational cohort study (*n* = 1,758) (15)	Retrospective analysis Consensus^e^
Risk Factors (n)	3	4	4	5	6	7	7	8
	1. Birth between 3 months before and 2 months after season start date 2. Smokers in the household and/or maternal smoking whilst pregnant 3. Siblings and/or daycare attendance	1. Birth ±10 weeks of season start 2. Mother smoking during pregnancy 3. School-age siblings or day care attendance 4. Sex	1. Born Aug 14th to Dec 1st 2. Presence of siblings or subject day care attendance 3. Breast fed ≤2months or not 4. Atopy in 1st degree family member	1. Birth between Aug 14th and Dec 1st 2. Day care attendance and/or siblings 3. Neonatal respiratory support 4. Breastfeeding ≤4 months 5. Maternal atopic constitution	1. Age on 1st October ≤3 months 2. Smoking among family members 3. Mother smoking or during pregnancy 4. Subject day care attendance 5. Children 4–5 years old present 6. Age of mother at delivery ≤25 years	1. Birth ±10 weeks of season start 2. Number of siblings ≥2 years 3. Sex 4. Birth weight 5. Breast feeding ≤2 months 6. Number of family members with atopy 7. Number of family members with wheeze	1. Born during RSV season (Nov-Jan) 2. >1 smoker in the household 3. Subject or siblings attending day care 4. >5 individuals in the home, including the subject 5. Sex 6. Family history without eczema 7. Small (<10th percentile) for GA	1. Born near or during RSV season (1st May to 31st March) 2. Passive smoking at home 3. Maternal smoking during pregnancy 4. Siblings <10 years 5. Nursery school attendance 6. No breastfeeding 7. Male sex 8. Surfactant in the first days of life
Sensitivity/Specificity	0.69/0.73	0.062/0.99	0.46/0.79	Low risk (1% RSVH): 0.90/0.35 High risk (13% RSVH): 0.32/0.90	NR	0.72/0.71	0.68/0.72	0.61/0.58
ROC AUC^g^	0.773	0.687	0.703	0.72	0.755	0.791	0.762	(0.618)^f^
Validation	Internal ROC AUC: 0.773^b^ External with Irish (5, 16), Colombian (17) & Brazilian (18) data	NR	Internal ROC AUC: 0.702^c^ Against separate validation cohort within RISK	Internal ROC AUC: 0.72^c^ Update and validation of RISK score (9)	NR	Internal ROC AUC: 0.785^b^ External with German, Italian (19), French (20), & Danish (21) data	External with Spanish data & Canadian prospective study (22)	Analysis compared with IRST
Risk score	Low risk: ≤19; Moderate risk: 20–45; High-risk: 50–56	Presence of all 4 risk factors	≥16: RSVH risk 10.0%; <16: RSVH risk 3.5%	Low risk: ≤4; Moderate risk: 5–7; High-risk: ≥8	NR	NR	Low risk: 0–48; Moderate risk: 49–64; High-risk: 65–100	Presence of ≥3 risk factors
Cost-effectiveness (Yes/No)	Y [Canada (23), Italy (24), Colombia (25), Korea (26)]	Y [Spain (27)]	N [Netherlands (28)]	Not assessed	Not assessed	Not assessed	Y [Canada (23, 29, 30)]	Not assessed

CRST, Canadian RST; FLIP, risk factors linked to respiratory syncytial virus infection requiring hospitalization in premature infants study; GA, gestational age; IRST, International RST; NR, not reported; PICNIC, pediatric investigators collaborative network on infections in Canada; PONI, predictors associated with RSV hospitalization in nonprophylaxed premature infants; RISK, [no acronym]; ROC AUC, area under the receiver operating characteristic curve; RST, risk scoring tool.

^a^ IRST combined data from FLIP-2 (13), RISK (9), PONI (11), PICNIC (15), Italian Birth Cohort (31) and REPORT [Respiratory Syncytial Virus (RSV) Respiratory Events Among Preterm Infants Outcomes and Risk Tracking Study] (32).

^b^ 100-fold bootstrapping.

^c^ 1,000-fold bootstrapping.

^d^ Twenty-three countries in Western Europe (Austria, France, Norway, Portugal, Sweden, and Switzerland), Eastern Europe (Bosnia, Bulgaria, Czech Republic, Estonia, Latvia, Lithuania, Slovakia and Slovenia) and Russia, South Korea, Mexico and the Middle East (Bahrain, Egypt, Jordan, Lebanon, Oman and Saudi Arabia).

^e^ Unclear from publication, appears to have been developed by consensus after a review of several guideline publications.

^f^ Accuracy derived from contingency tables.

^g^ ROC curves are constructed by plotting the sensitivity (true positives; number of RSV hospitalized infants predicted to be hospitalized) against the specificity (false positives; number of non-hospitalized infants predicted to be RSV hospitalized), with areas closer to one representing better predictive accuracy.

## Robustness and applicability of source data

The majority of published RSTs and predictive models have been developed from large, prospective, observational studies specifically designed to identify risk factors for RSVH in moderate-to-late preterm infants (Table 1; 5–11). Studies include those from Canada [PICNIC (*n* = 1,758) (15)], the Netherlands [RISK (*n* = 2,421) (9) and RISK-II (*n* = 1,564) (10)], and Spain [FLIP (*n* = 554) (14), FLIP-2 (5,441) (13)], all of which have been used to develop country-specific RSTs (Table 1) (6–10). The IRST was developed using pooled data from the PICNIC, RISK and FLIP-2 studies as well as evidence from Italy [Italian Birth Cohort (*n* = 2,210) (31)], the USA [REPORT (*n* = 1,642) (32)], and an international study involving 23 countries predominantly from Europe but also having representation from Asia, the Middle East and Latin America [PONI (*n* = 2,390) (11)] (5). In total, the dataset underpinning the IRST included risk factor data on 13,475 infants of which 484 (3.6%) had a confirmed RSVH (5).

This raises the important point that any RST for predicting RSVH risk should be derived from data on moderate-to-late preterm infants with confirmed RSV infection (either through antigen or PCR testing) and should not be based on a clinical diagnosis of suspected RSV bronchiolitis. Developing an RST or predictive model using cases of presumptive RSV infection undermines the validity of an infant's predicted risk for RSVH and the overall rationale of the RST for guiding RSV prophylaxis. In addition, this strongly implies that the data used to develop the RST should exclude subjects who received RSV prophylaxis, as this would pollute the categorization of infants with and without RSVH. For the IRST, only studies where ≤15% of infants received RSV prophylaxis were included in the pooled dataset and all such recipients were excluded from analysis (5).

## Balancing simplicity and accuracy

There are several risk factors that have been significantly associated with an increased risk of RSVH in moderate-to-late preterm infants that can be selected for inclusion within an RST. In the eight RSTs/predictive models summarized in Table 1, a total of 15 distinct risk factors were used. The most common risk factor, present in all eight RSTs/predictive models, is age relative to the RSV season, which is perhaps unsurprising as it is well recognized that RSVH risk increases with decreasing chronological age. Two further risk factors, present in seven RSTs/predictive models, relate to crowding and viral spread, specifically: presence of siblings and attendance at daycare. The next most frequently included risk factors are smoking (during pregnancy and/or in the household), lack of breastfeeding, and familial atopy, all of which are part of four RSTs/predictive models.

The number of risk factors that comprise the eight RSTs/predictive models ranges from three to eight, with the most predictive one, developed from the Spanish FLIP study (SFRST; 14), incorporating seven variables (6). Despite the high predictive accuracy of this RST [area under the receiver operating characteristic curve (AUROC) 0.791 (6)], it could be argued that assessing seven risk factors for a child is somewhat unwieldy, particularly when four of them are continuous (parametric) rather than simple dichotomous or categorical variables and one (breast feeding) cannot be explicitly verified. It is for this reason that the IRST was intentionally developed to include as few as possible categorical risk factors—winnowing 18 variables down to three (relating to age, smoking and siblings/daycare)—whilst maintaining a high level of predictive accuracy (AUROC 0.773) (5).

A critical decision for any RST is the cut-off level or score for identifying high-risk infants, apart from categorizing those who are at low- and moderate-risk for RSVH. For the SIN_LAZIO_ score, this was accomplished by assigning high-risk to any infant with ≥3 of the eight included risk factors (12). Infants with ≥3 risk factors were found to have a 2.2 greater risk of non-specific viral bronchiolitis than those with <3 risk factors (12). For the IRST and Dutch RST (DRST; RISK-I/II), the low-risk group was set at a RSVH rate of 1%, with the moderate- and high-risk groups dichotomized by plotting the RSVH rate against the risk score and selecting a point of natural inflection (5, 10). The Canadian RST (CRST) used a slightly different approach by identifying the point of highest accuracy for differentiating two populations—the low- and combined moderate- and high-risk categories—and then defining the high-risk group after review of scoring frequency (8). The average RSVH rate in the high-risk category was approximately twice as high with the CRST than the IRST (18.7% vs. 9.5%, respectively), with the DRST being intermediate between the two RSTs (13%) (5, 8, 10).

These varied approaches to defining cut-off scores have implications for the proportion of infants classified in the moderate- and high-risk groups who would ultimately be eligible for palivizumab prophylaxis. The proportion of the respective populations assigned high-risk was 11% with the DRST (10), 23.6% with the IRST (5), and 41.6% with the SIN_LAZIO_ score (12). This proportion was not reported for the CRST. However, a subsequent report comparing the CRST and IRST with a standardized population established that while a similar percentage of infants were categorized as high-risk (0.6% vs. 0.7%, respectively), a far larger proportion of infants were classified as moderate-risk by the IRST (19.9% vs. 9.8% by the CRST) (33). A further prospective study from Canada recently documented that 4.9% of infants were scored as high-risk, based on the IRST (34). These latter results highlight that the proportion of infants assigned to a risk category varies depending on the study design, the population being tested specifically for RSV and therefore the importance of validation exercises.

## Validation and applicability

In compliance with best practice, a RST should be robustly validated prior to adoption in order to lend credence to the underlying predictive model. The IRST, SFRST, DRST and Dutch-RISK RST were all internally validated using a bootstrapping approach wherein 100–1,000 copies of the source dataset were created using sampling with replacement and the average predictive accuracy (with dispersion) calculated across these datasets (Table 1; 5, 6, 9, 10). For all four RSTs, bootstrapping confirmed the models were internally consistent and not overly optimistic (i.e., there was little or no over-fitting) (5, 6, 9, 10). For the IRST, the mean AUROC from bootstrapping was identical to that derived from the original source data (both 0.773) (5).

The true test of an RST is validation against an external database or population. The SIN_LAZIO_ score was assessed using retrospective data on Italian moderate-to-late preterm infants with (20% RSV+) and without bronchiolitis (12). The SFRST was validated against several databases of moderate-to-late preterm infants with and without confirmed RSVH, including those from Germany (6), Italy (19), France (20), and Demark (21); supporting its applicability in European populations. The DRST was originally derived from the RISK study [which was informed by the SFRST (6)] and then prospectively validated and updated by the RISK-II study (9, 10); thus, demonstrating its applicability to the Dutch population. As for the CRST, this was first validated against the Spanish FLIP study before being tested prospectively in routine clinical practice in Canada (8, 22). In the prospective validation, 78 (18.1% of 430) infants at moderate- and high-risk, as scored by the CRST, received palivizumab and the RSVH rate was low at 1.6% (22). This strongly supported the utility of the CRST in Canadian infant population. The IRST was first validated against the RSV Preterm Risk Estimation Measure for RSVH in Ireland (PREMI) study (5, 16) before further validations were undertaken using Brazilian (18) and Colombian (17) data. Taking into consideration that the IRST was developed using data from six studies [including the multinational PONI study (11) that included data from 23 countries] the subsequent validations strongly establish its universal reproducibility and generalizability to new and different ethnic populations.

## Cost-effectiveness

Ultimately, whether an RST is worthwhile deploying in a country is dependent on its ability to guide palivizumab prophylaxis cost-effectively (vs. no prophylaxis). The CRST, DRST and FLIP-2 model have all been assessed in cost-utility analyses for their respective countries, with risk factor-guided prophylaxis proving cost-effective in Canada (23, 29, 30) and Spain (27), but not the Netherlands (28). Perhaps unsurprisingly, RST-guided palivizumab prophylaxis was recommended in Spain (35) and certain provinces of Canada (36, 37), but not the Netherlands (38).

The IRST has been found to guide palivizumab prophylaxis cost-effectively in several continents and economies, including North America [Canada (23)], Europe [Italy (24)], Latin America [Columbia (25)] and Asia [South Korea (26)], and its use is recommended in International Consensus guidelines (39). A salient difference between the FLIP-2, CRST and IRST economic studies and the Dutch report is that the former modelled respiratory morbidity for 6–18 years, whereas the latter used a 1-year time horizon (23, 27–30). It is now well-established that respiratory morbidity can persist throughout childhood (40, 41) and has been reported to be a key driver of palivizumab cost-effectiveness (23, 24). It would be interesting to investigate whether DRST-guided palivizumab prophylaxis achieves cost-effectiveness in the Netherlands healthcare system if respiratory morbidity was modelled for 6 years or longer.

The IRST and CRST were both assessed in the Canadian healthcare system using the same cost-utility model and, while palivizumab was found highly cost-effective using both RSTs, the incremental cost-utility ratio (ICUR) was lower in the latter (CAN$29,789 vs. CAN$15,833, respectively) (23). This might lead one to conclude that the CRST should be the preferred option for use in Canada. However, the IRST can be considered simpler (3 risk factors vs. 7 for the CRST) and, importantly, covers more potential RSVHs (85% vs. 54%) (23, 33).

## Discussion

We strongly believe that moderate-to-late preterm infants should be protected from both the shorter- and longer-term burdens of RSV infection. RSTs provide an evidenced-based approach for cost-effectively guiding palivizumab prophylaxis towards moderate-to-late preterm infants who are most at-risk for RSVH. When considering the various attributes of the published RSTs and predictive models, the IRST combines simplicity with a high level of predictive accuracy for RSVH and its cost-effectiveness has been well-demonstrated in multiple countries and economies. For those countries with no or limited use of palivizumab in moderate-to-late preterm infants, adoption of the IRST can support reimbursement following local validation and ensure, with a well-established degree of precision, that the most vulnerable of these infants receive prophylaxis.
